# Molecular Determinants of Neutrophil Extracellular Vesicles That Drive Cartilage Regeneration in Inflammatory Arthritis

**DOI:** 10.1002/art.42958

**Published:** 2024-08-16

**Authors:** Bethan L. Thomas, Trinidad Montero‐Melendez, Silvia Oggero, Magdalena K. Kaneva, David Chambers, Andreia L. Pinto, Alessandra Nerviani, Davide Lucchesi, Shani Austin‐Williams, Mohammed T. Hussain, Costantino Pitzalis, Benjamin Allen, Marzia Malcangio, Francesco Dell'Accio, Lucy V. Norling, Mauro Perretti

**Affiliations:** ^1^ Queen Mary University of London London United Kingdom; ^2^ Queen Mary University of London and Kings College London, Guys’ Campus London United Kingdom; ^3^ Kings College London, Guys’ Campus London United Kingdom; ^4^ Royal Brompton & Harefield NHS Foundation Trust London United Kingdom; ^5^ Queen Mary University of London, Barts Health NHS Trust, and National Institute for Health and Care Research Barts Biomedical Research Centre London United Kingdom

## Abstract

**Objective:**

This study was undertaken to establish the potential therapeutic profile of neutrophil‐derived extracellular vesicles (EVs) in experimental inflammatory arthritis and associate pharmacological activity with specific EV components, focusing on microRNAs.

**Methods:**

Neutrophil EVs were administered intra‐articularly through a prophylactic or therapeutic protocol to male C57BL/6 mice undergoing serum‐transfer–induced inflammatory arthritis. Transcriptomic analysis of knees was performed on joints following EV administration, naive and arthritic mice (untreated; n = 4/group) and EV‐treated diseased mice (intra‐articular administration) with contralateral (vehicle‐treated; n = 8/group). Comparison of healthy donor and patients with rheumatoid arthritis (RA) neutrophil EVs was performed.

**Results:**

EVs afforded cartilage protection with an increase in collagen‐II and reduced collagen‐X expression within the joint. To gain mechanistic insights, RNA sequencing of the arthritic joints was conducted. A total of 5,231 genes were differentially expressed (*P* < 0.05), with 257 unique to EV treatment. EVs affected key regenerative pathways involved in joint development, including Wnt and Notch signaling. This wealth of genomic alteration prompted to identify microRNAs in EVs, 10 of which are associated with RA. As a proof of concept, we focused on miR‐455‐3p, which was detected in both healthy donor and RA EVs. EV addition to chondrocyte cultures elevated miR‐455‐3p and exerted anticatabolic effects upon interleukin‐1β stimulation; these effects were blocked by actinomycin or miR‐455‐3p antagomir.

**Conclusion:**

Neutrophils from patients with RA yielded EVs with composition, efficacy, and miR‐455‐3p content similar to those of healthy volunteers, suggesting that neutrophil EVs could be developed as an autologous treatment to protect and repair joint tissue of patients affected by inflammatory arthritides.

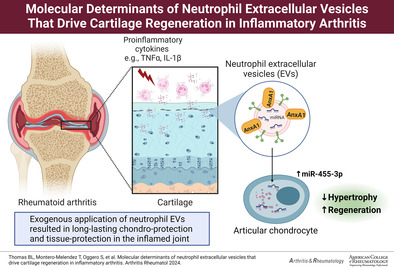

## INTRODUCTION

There is need for novel therapeutic approaches for treating rheumatoid arthritis (RA). Despite a plethora of successful RA therapies with different modes of action, most patients with RA are not in remission, 10%–15% are refractory to treatment, and there remains no cure.^1^, ^2^, ^3^ Moreover, biologics can cause immunosuppression and promote generation of antidrug antibodies, limiting their benefit.^4^, ^5^ Additionally, these therapies afford minimal tissue repair and disease may progress to impose patient joint replacement, an approach that may have limited life span and require revision surgery.

Over the last decade, neutrophils have emerged as orchestrators of the resolution of inflammation. Thus, aside from proteolytic enzymes and reactive oxygen species, neutrophils can also synthesize proresolving mediators such as lipoxin A_4_ and annexin A1 (AnxA1).^6^, ^7^ Furthermore, as originally shown by Gasser and Schifferli,^8^ neutrophils release extracellular vesicles (EVs) that display anti‐inflammatory and tissue‐protective properties.^9^, ^10^ In human RA synovial fluids, we have described an AnxA1^+^ EV subset that promotes cartilage repair ex vivo.^11^ EVs released from human neutrophils stimulated with tumor necrosis factor alpha (TNF‐α) penetrate cartilage to promote anabolic responses.^11^ In the same study, interfering with endogenous EV production through a genetic approach worsened cartilage disruption in experimental arthritis, though this effect could not be pinpointed to neutrophil EV specifically. Herein, we aimed to define the duration of tissue‐protective properties of neutrophil EVs and identify molecular determinants that could underpin such a biological property. We focused on microRNAs as potential effectors of EV responses and identified microRNA (miR)‐455‐3p as a mediator that activates tissue‐protective circuits in chondrocytes and cartilage.

## METHODS

### Isolation and characterization of blood neutrophils and their EVs

Blood was collected from healthy volunteers as approved by the Queen Mary Ethics of Research Committee (QMREC2014.61) and from patients diagnosed with RA (American College of Rheumatology/EULAR 2010 classification criteria)^12^ as part of the Research Tissue Bank (REC Ref. No. 17/WS/0172). Neutrophils were isolated using a double histopaque method^11^ and stimulated (2 × 10^7^ cells/mL in phenol red‐free RPMI) with 50 ng/mL TNFα for 20 minutes at 37°C for EV generation. In our previous study, we demonstrated that TNF‐α activation of neutrophils yields a higher number of EVs than those enumerated from nonstimulated control cells; the proportion of vesicles positive for CD66b, AnxV, phalloidin, and myeloid‐rich protein 14 and 8 did not differ, yet the proportion of AnxA1 positive vesicles are higher along with the total AnxA1 expression—more closely mimicking the RA patient phenotype.^11^ The suspension was centrifuged (4,400*g* for 15 minutes then 13,000*g* for 2 minutes at 4°C) to remove cells and apoptotic bodies. Finally, supernatants were ultracentrifuged (100,000*g* for 70 minutes at 4°C) and pellet resuspended in phosphate buffered saline (PBS) at 5 × 10^7^/100 μL. EV concentration and size was determined with Nanosight NS300TM using the same detection threshold for each sample and ensuring the particle count was between 30 and 70 per frame. Characterization of EV markers and uptake by chondrocytes was performed by ImageStream and Western blotting; immunogold labeling and transmission electron microscopy of EVs are detailed in Supplementary Methods.

### Isolation and culture of human articular chondrocytes and cell lines

Human patient samples were obtained under Rec N.07/Q0605/29. Adult human articular cartilage was obtained from patients undergoing joint replacement for knee osteoarthritis (OA) after obtaining informed consent. Primary adult human articular chondrocytes and C28/I2 cell line were expanded in Dulbecco's modified eagle medium/nutrient mixture F‐12 with 10% fetal bovine serum (FBS) (Gibco). For experimentation, FBS was replaced with 1× insulin‐transferrin‐selenium supplement (Gibco).

### KBxN serum‐transfer–induced arthritis

All animal procedures were approved by the local ethics committee and the UK Home Office under licenses PPL70/8264 and PPL70/7986. Male 10‐week old C57BL/6 mice were injected with 100 μL of arthritogenic serum at days 0 and 2 (and days 13 and 24 if the experiment was extended past 10 days). The development of arthritis was assessed daily, and the severity of arthritis was scored for each paw on a three‐point scale, in which 0 = normal appearance, 1 = localized edema over one part of the paw, 2 = edema involving more than one part of the paw (eg, digit and ankle), 3 = marked edema involving the whole paw (ie, digits, ankle, and pad. The scores of all four paws were added for a composite score, with a maximum score of 12 per mouse. Knee thickness was measured using a digital caliper. EV treatment was given intra‐articular at the reported doses in 5 μL on day 3 of serum‐transfer–induced arthritis (STIA), except for therapeutic treatment experiment, when they were given on day 20. An incapacitance test was used to measure the weight bared between the hind limbs of the mice to compare EV‐treated versus contralateral control limb. Histological analysis of tissues is included in Supplementary Methods.

### Cartilage formation assay in nude mice

The ectopic cartilage formation assay was conducted as described.^13^, ^14^ Freshly isolated human articular chondrocytes (1 × 10^6^, passage 1) were mixed with 1.0 × 10^5^ EVs or PBS vehicle and collagen type‐1 to form 100 μL final suspension, which was injected subcutaneously onto the backs of female CD1^nu/nu^ mice. Cartilage nodules were harvested two weeks after implantation.

In total, 96 mice were used for the study. Mice were excluded if they lost 20% of their body weight from the start of the experimental period. A different investigator was responsible for preparing animal treatments (and was the only researcher aware of treatment group allocation) to those performing outcome assessment and data analysis. RNA extraction, sequencing, and analysis by quantitative real‐time polymerase chain reaction are detailed in Supplementary Methods.

### microRNA arrays

microRNA were extracted from isolated EV preparations using the miRNA Serum Plasma Kit (Qiagen). The composition of the isolated RNA populations was determined using mRNA Pico bioanalyzer profiling. The complexity of the microRNA cohorts was surveyed using Thermofisher miRNA 4.1 arrays in conjunction with the Flashtag labeling system. The list of expressed microRNAs was determined by statistical processing using the RMA‐DABG preprocessing package embedded in TACX software (Thermofisher).

### In vitro miR blocking experiment

Antagomir experiments to block the function of miR‐455‐3p were performed using mirVana® microRNA inhibitor mir‐455‐3p (Assay ID MH11142; Thermofisher AM17000) and Anti‐miR miRNA Inhibitor Negative Control #1 (Thermofisher AM17010). Antagomirs were transfected into the cells using jetPRIME reagent (Polyplus). After 48 hours, cells were stimulated with interleukin (IL)‐1β (20 ng/mL) with or without EVs for a further 24 hours.

In separate experiments, human chondrocytes were pretreated with actinomycin D (2 mg/mL) for 30 minutes before the addition of EVs (3 × 10^7^). The expression of miR455‐3p within the chondrocytes was quantified after 24 hours.

### Data representation and statistics

All data are expressed as mean ± SD or as paired analysis where contralateral internal controls are available. All data are assessed using nonparametric statistical tests unless otherwise stated. Comparisons of two groups are assessed using nonparametric *t*‐test (Mann‐Whitney U), or nonparametric paired *t*‐test (Wilcoxon signed rank test) in which comparisons are made between treated and contralateral limbs of the same mouse. Where more than two treatment groups are present, data were assessed with a nonparametric one‐way analysis of variance (ANOVA; Kruskall Wallis test) with multiple comparisons (Dunn's multiple comparison). In Figure S2D, in which data were modeled using a two‐way ANOVA, a quantile‐quantile plot was used to assess that the residuals approximated a normal distribution. Specific tests and number of replicates for each experiment have been outlined in figure legends.

## RESULTS

### Neutrophil EVs afford long‐lasting cartilage protection

Neutrophil‐derived EVs were generated from healthy donors as depicted in the schematic (Figure S1A) and displayed the expected characteristic EV markers as determined by Western blotting (Figure S1B). The size (median diameter approximately 120 nm) and number of EVs generated was not modulated by donor age or sex (Figure S1C–F). Their heterogenous size was confirmed by electron microscopy (Figure S1G), and AnxA1 presence by Western blotting (Fig S1B) and immuno‐gold labeling (Figure S1H).

We started by confirming that intra‐articular injection of 0.3 × 10^5^ EVs generated from healthy donor neutrophils at day 3 afforded chondroprotection 48 hours later, at day 5 of the STIA model (Figure S2A and B).^11^ In a dose escalation analysis (0.3–3.0 × 10^5^ EVs per joint) with outcome measured on day 10 (7 days postadministration), 1.0 × 10^5^ EVs afforded the greatest protection against proteoglycan loss, compared to vehicle‐treated contralateral joints (Figure S2C–E). Intra‐articular administration of EVs preserved expression of collagen type‐II (a major component of the articular cartilage) in five out of eight mice with a concomitant and significant decrease in collagen type‐X (marker of chondrocyte hypertrophy) positive chondrocytes (Figure S2F–H). On these bases, we selected the 1.0 × 10^5^ EV dose for subsequent experiments.

**Figure 1:**
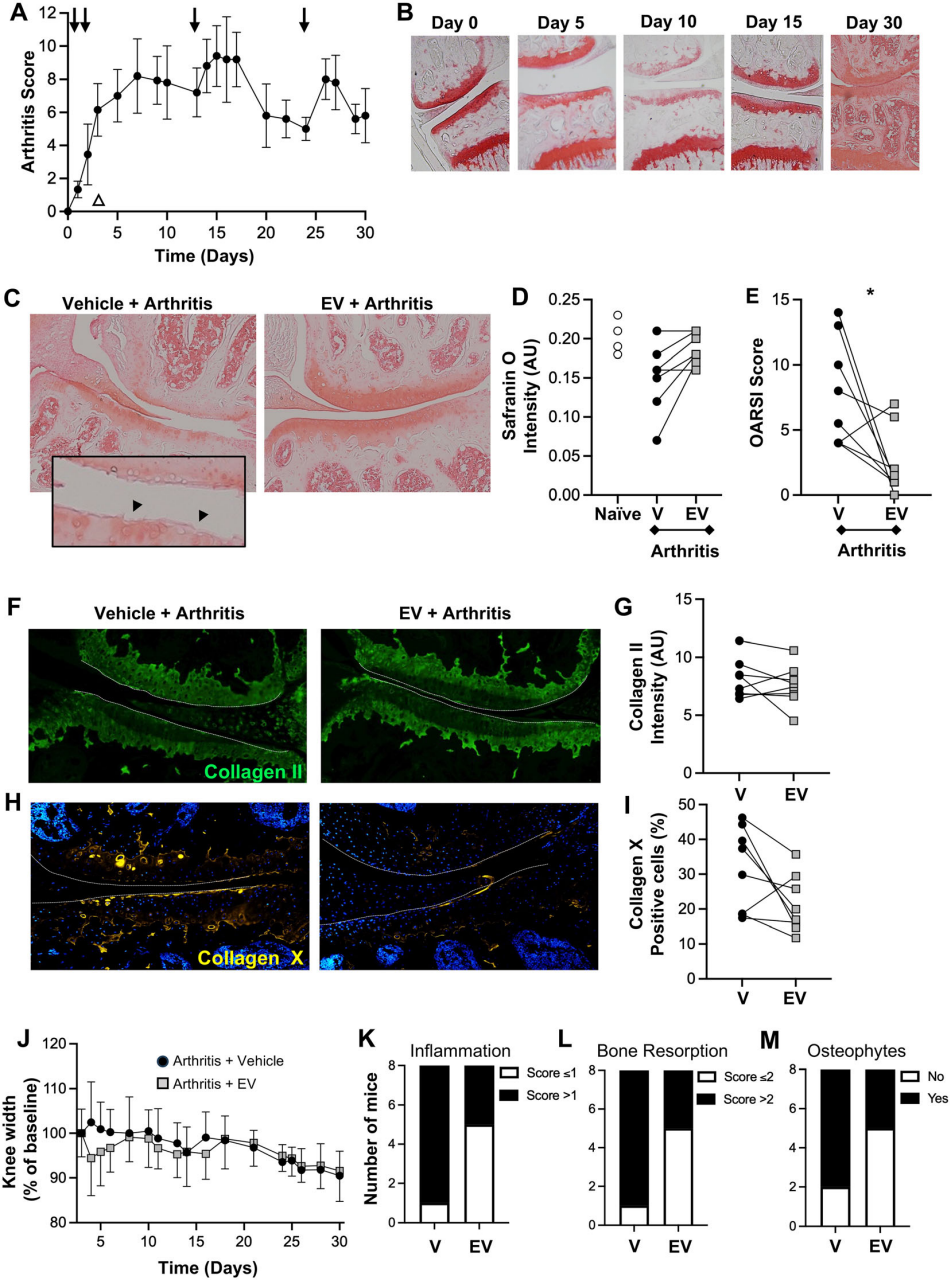
Long‐term chondroprotective properties of neutrophil EVs. K/BxN serum was injected i.p. at day 0, 2, 13, and 24 (arrows) to induce arthritis (STIA). Mice were treated on day 3 intra‐articularly with 1.0 × 10^5^ EV in one knee and vehicle in contralateral knee (open triangle) and disease profile was monitored over 30 days (A and B). (A) Disease profile (score out of 12 for inflamed digits, paws, and wrists). Data are mean ± SD, 5 mice per group. (B) Characterization of cartilage breakdown from vehicle‐treated knees at indicated timepoints of the protocol shown in panel A; representative images of safranin O‐stained knee joints, used to measure proteoglycan content. (C) Day 30 safranin O‐stained images of arthritic mice treated with vehicle or EVs and (D) quantification and (E) OARSI score (sum of all compartments) for structural integrity (*P < 0.05 nonparametric *t*‐test, Wilcoxon matched‐pairs). (F–I) Day 30 staining for collagen type‐II and collagen type‐X, expressed as intensity within the extracellular matrix and percentage of total (DAPI+) cells. Data are mean ± SD, n = 8 mice per group. (J) STIA was conducted as in A, knee width measurements in EV‐treated (EV) and contralateral vehicle‐treated (V) measured as percentage of baseline at day 3. Data are mean ± SD, n = 8 mice per group. (K–M) Day 30 histological scores for inflammation (0–3), bone resorption (0–5), and osteophyte formation. EV, extracellular vesicle; i.p., intraperitoneal; OARSI, Osteoarthritis Research Society International; STIA, serum‐transfer–induced arthritis.

To ascertain whether neutrophil EVs sustained long‐lasting effects, arthritis was monitored for 30 days, with booster injections of arthritogenic serum on days 13 and 24 to extend the duration of arthritis (Figure 1A). In the arthritic joint, loss of cartilage proteoglycans was detected as early as day 5, and this effect was protracted until day 30 (Figure 1B). However, a single injection of 1.0 × 10^5^ EVs at day 3 afforded local cartilage protection for up to 30 days. Treatment with EVs protected cartilage, reduced proteoglycan loss, and preserved tissue structural integrity as analyzed with the Osteoarthritis Research Society International (OARSI) score^15^ (Figure 1C–E). On day 30, seven out of eight mice displayed less collagen type‐X positive cells in EV‐treated knees, whereas no difference was quantified for collagen type‐II (Figure 1F‐I). Joint swelling increased after arthritogenic serum, with modest attenuation after administration of EVs, evident only up until day 5 (Figure 1J). Histological scoring of arthritis revealed a significant difference in the extent of joint damage (scoring method in Figure S3), with EV‐treated joints presenting a lower total score, with only three out of eight mice reaching a score ≥1. In the contralateral knees, seven out of eight mice displayed histological score ≥1 (Figure 1K). Similar results were obtained for bone resorption, with EV‐treated joints presenting less bone resorption than vehicle‐treated contralateral joints (Figure 1L). We also assessed each joint for presence of osteophytes: although osteophyte formation occurred during the model, the difference between treatment groups was less apparent (Figure 1M).

### Effect of neutrophil EVs on pain

To test whether neutrophil EVs could mitigate pain‐related symptoms, an incapacitance test was performed to measure body weight distribution between hind limbs. A short‐term assessment two and four days postpharmacological treatment (0.3 × 10^5^ EVs administered intra‐articularly on day 3) revealed no significant difference in weight bearing between EV or vehicle‐treated contralateral knees (Figure S4A). When we studied the association between weight‐bearing distribution and inflammatory score for each hind limb, irrespective of treatment, an inverse correlation emerged, indicating that any change in weight bearing was related to local inflammation levels (Figure S4B). This lack of EV modulation of pain‐related behavior was supported at tissue level, because no change in calcitonin gene‐related peptide expression (a marker of nociceptive neurons) in the lumbar dorsal root ganglia was quantified (Figure S4C and D).

### Therapeutic relevance of neutrophil EVs

**Figure 2:**
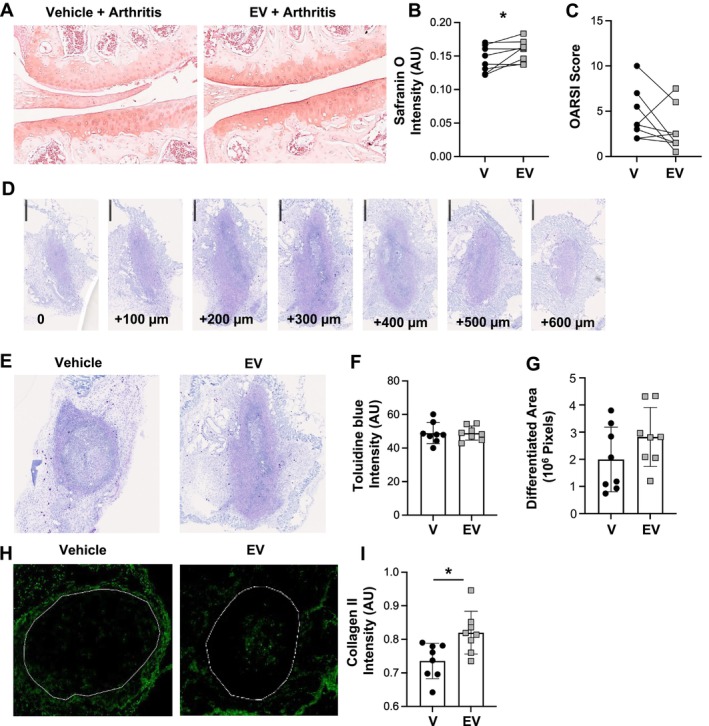
Therapeutic and translational effects of neutrophil EVs. (A and B) K/BxN STIA was induced as in Figure 1A. Mice were treated intra‐articularly with 1.0 × 10^5^ EV (EV) or PBS vehicle (V) on day 20 and experiment terminated at day 30. (A) Representative images for safranin O staining of knee sections. (B and C) Quantitative data for safranin O staining within the knee sections and OARSI score for structural integrity of knee section (*P < 0.05 nonparametric *t*‐test, Wilcoxon matched‐pairs). (D–I) Human articular chondrocytes translation model. Chondrocytes (1 × 10^6^) were mixed with a collagen type‐I scaffold gel and either 1.0 × 10^5^ EV (EV) or vehicle (V) before subcutaneous injection onto six‐week‐old nude mice. (C) Two weeks following implantation, cartilage nodules were harvested and sectioned through at 100‐μm intervals before toluidine blue staining. Images for representative nodule shown. (D) Analyses of the central sections of the nodules for intensity of the proteoglycan stain and size of the differentiated cartilage area and representative images and cumulative data. For each single nodule three central sections were used in the analysis. Scale bar = 500 μm. (E) Representative images for collagen type‐II staining within the nodules and data for staining intensity. Cumulative data are mean ± SEM, n = 8 mice per group. *P < 0.05, Mann‐Whitney test. EV, extracellular vesicle; OARSI, Osteoarthritis Research Society International; PBS, phosphate buffered saline; STIA, serum‐transfer–induced arthritis.

Having described the protection afforded by EVs upon a prophylactic protocol, the effect of EVs on restoring cartilage integrity following a therapeutic protocol was tested. Because cartilage damage was evident at day 20 (Figure 1B), 1.0 × 10^5^ EVs were administered at this timepoint and analyses run at day 30 (10 days posttreatment). In these settings, EV‐treated joints displayed significantly higher levels of proteoglycans within the cartilage extracellular matrix (Figure 2B), together with a trend toward improved structural integrity using the OARSI score (Figure 2C).

To further investigate the translational potential for neutrophil EVs as a viable therapy, we next established efficacy in human tissue using a validated murine transplant model,^13^, ^14^ in which the formation of human cartilage nodules in an in vivo setting can be quantified. A scaffold containing human chondrocytes mixed with 1.0 × 10^5^ EVs or PBS vehicle was injected subcutaneously into CD1^nu/nu^ mice. Histological analyses of the central sections of cartilage matrix molecules two weeks postimplantation (see Figure 2D) indicated no difference in proteoglycan content (Toluidine blue staining) or size of the differentiated area (Figure 2E–G). However, collagen type‐II content was significantly higher in EV‐treated nodules compared to controls (Figure 2H and I), indicating EV display anabolic properties on human chondrocytes in vivo. Altogether these data justified a deeper investigation on the molecular determinants responsible for the reparative and regenerative properties of neutrophil EVs.

### Neutrophil EV AnxA1 is required for uptake into chondrocytes

We previously reported a functional role for AnxA1+ EV to enable ex vivo cartilage penetration.^11^ To investigate the potential involvement of this marker in our settings, human C28/I2 chondrocyte cells were incubated with fluorescently labeled EVs derived from neutrophils from healthy donors. Confocal microscopy revealed presence of adherent EVs to the surface of chondrocytes after six‐hour incubation (Figure S5A), and ImageStream demonstrated vesicle internalization (green staining; Figure S5B). Addition of an anti‐AnxA1 blocking antibody reduced EV‐associated fluorescence with human chondrocytes by approximately 30% to 40% on net values (Figure S5B and C).

Although this dataset confirms AnxA1 as a critical factor that mediates EV anchoring onto the chondrocyte, we hypothesized the involvement of other determinants to explain the potent and long‐lasting modulatory properties of EVs on joint cartilage. To address this, RNA sequencing analyses on vehicle‐ and EV‐treated knees of arthritic mice were conducted.

### Neutrophil EVs induce transcriptional changes in arthritic joints

**Figure 3:**
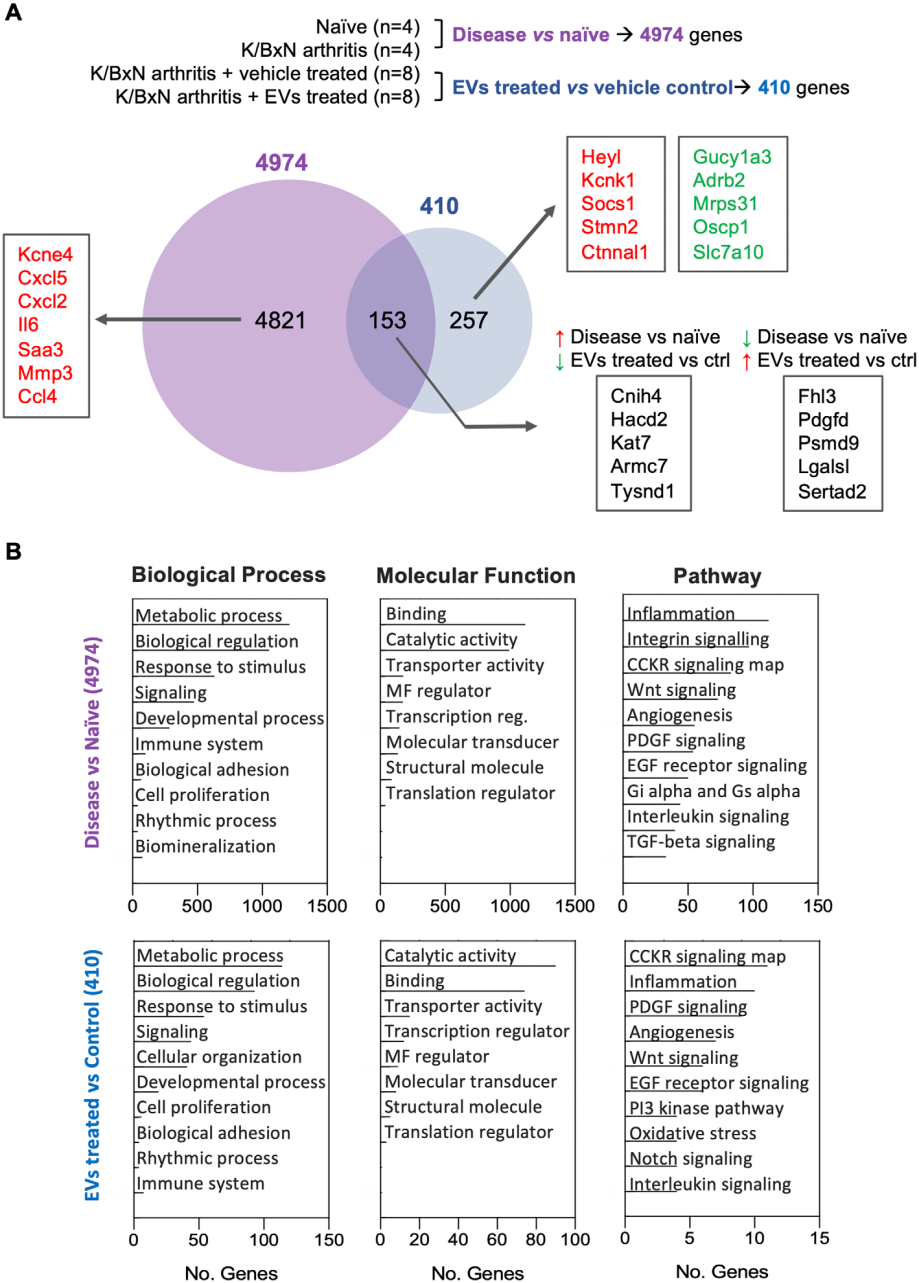
Transcriptomic analysis of EV‐induced changes in the joint cartilage. STIA mice were treated with either 1.0 × 10^5^ EVs or vehicle intra‐articular at day 3. At day 10, tibial and femoral cartilage surface, together with the subchondral bone, was harvested and RNA was extracted for sequencing. (A) Experimental design included nonarthritic (naive) and diseased (arthritic) mice (untreated), n = 4 knees/group, and EV‐treated diseased mice (intra‐articular administration) with contralateral control (vehicle‐treated) knees, n = 8 knees/group (paired). Venn diagrams presenting the total number of significantly altered genes in each comparison group. Groups were compared as disease vs naive or EV‐treated vs contralateral vehicle control: 153 genes altered by EV treatment were also altered by disease, whereas 257 genes were unique to EV treatment. Significantly altered genes were ranked by greatest fold change, and the top genes are displayed for each comparison group. For the genes regulated by disease and also counterregulated by the treatment, the top differentially regulated genes (up‐regulated in treatment with a significantly regulated decrease in disease or vice versa) have been displayed. (B) Functional analysis of processes, functions, and pathways for each of the comparisons (disease vs naive and EV‐treated vs nontreated) was conducted using Panther Classification System. CCKR, cell cycle‐related kinase; EGF, epidermal growth factor; EV, extracellular vesicle; MF, molecular function; PDGF, platelet‐derived growth factor; STIA, serum‐transfer–induced arthritis; TGF, transforming growth factor.

**Figure 4:**
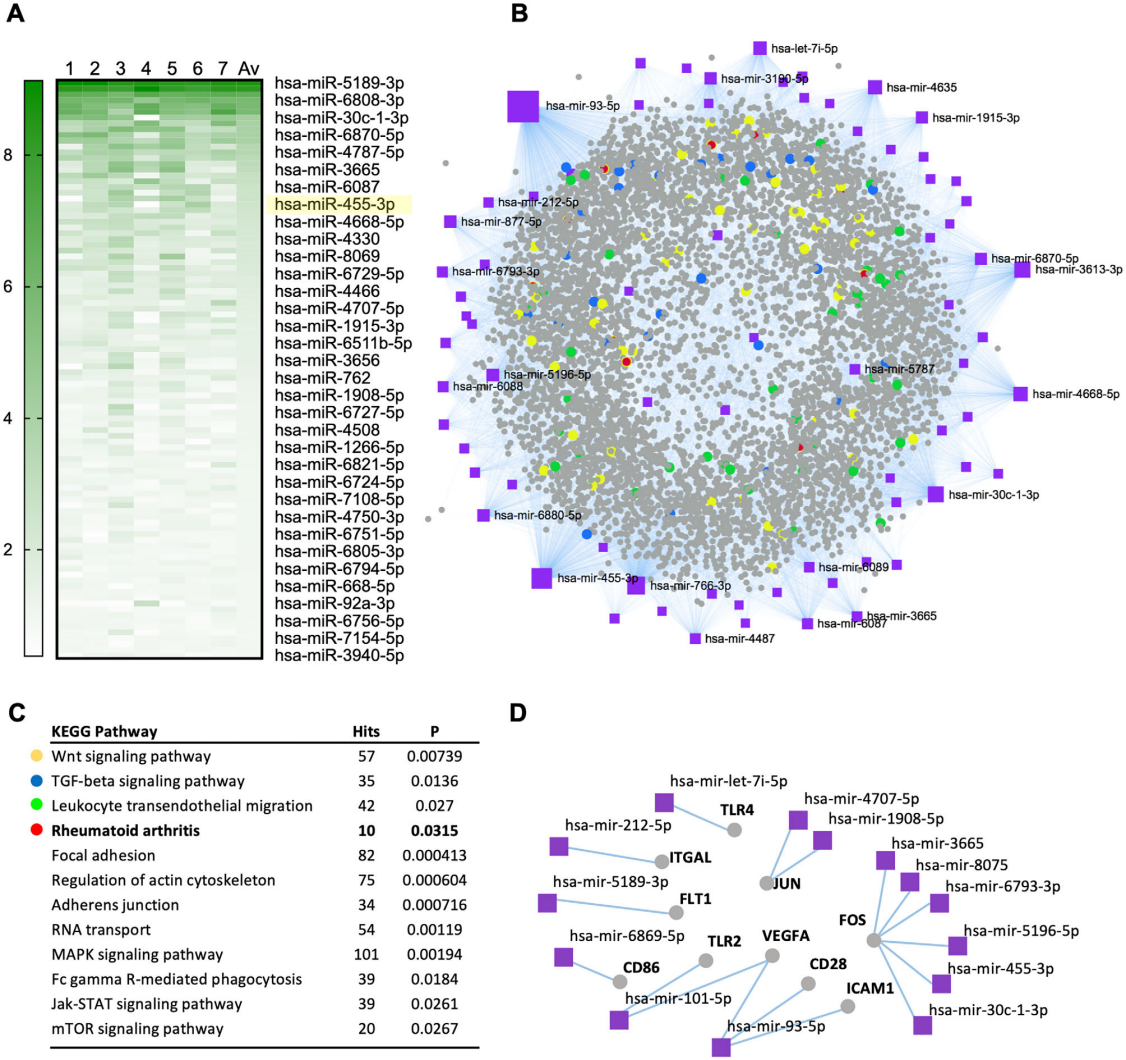
Investigation of miR content within neutrophil EVs. Seven distinct preparations of neutrophil EVs were analyzed using Thermofisher miRNA 4.1 GeneChip. (A) Heat map of the top 100 miRs (listed from higher‐to‐lower) shows consistency across donors. (B) Most abundant miR (ie, expression >1) within the EVs form a network with their target genes (network visual analytics platform miRNet 2.0). (C) Enriched pathways from panel B: colors are matched to highlight specific pathway involved in joint repair and disease. (D) Target genes related to rheumatoid arthritis were separated from panel B and displayed in a network in association with their targeting miRs which have been detected within the EVs. EV, extracellular vesicle; KEGG, Kyoto Encyclopedia of Genes and Genomes; MAPK, mitogen‐activated protein kinase; miR, microRNA; mTOR, mechanistic target of rapamycin; TGF, transforming growth factor.

EVs (1.0 × 10^5^) were administered intra‐articularly to arthritic mice on day 3, and knees were harvested at day 10 (day 7 posttreatment; Figure 3A). The experimental design allowed two separate analyses: arthritic animals with no treatment (disease) versus naive, and EV‐treated versus contralateral vehicle‐treated knees of arthritic mice. After normalization, background correction (transpopulation mapping > 10 counts) and statistical filtering (*P* < 0.05; see Supplementary Methods), a total of 5,231 genes were found to be differentially expressed in at least one of the comparisons, with 4,974 genes differentially expressed between disease versus naive and 410 genes between EV‐ and vehicle‐treated knees. Of these 410 hits, 257 were unique to EV treatment, whereas 153 gene products had been altered also by disease. We identified 27 of these genes to be symmetrically modulated by EV treatment (ie, increased in disease and down‐regulated by treatment or vice versa), indicating a direct rescue effect of the vesicles. Figure 4A presents the genes with the greatest change. A list of all the top gene changes can be found in Supplementary Tables (Table S1–S5). The full dataset of the RNA sequencing experiment has been deposited at GEO Database, with accession number GSE194152.

Pathway analysis identified an enrichment in genes associated with inflammatory response (like interleukins and chemokines) and cartilage matrix breakdown (eg, matrix metalloproteinases) (Figure 3B). Following EV treatment, we observed no significant regulation of those genes classically associated with cartilage matrix turnover, whereas signal transduction and cellular response gene categories were significantly changed (Figure 3; Table S1–S5). Specifically, EVs affected key pathways involved in limb and joint development, including Wnt and Notch signaling (Figure 3B). Altogether, these data suggest that treatment with neutrophil EVs genuinely activates regenerative responses within the tissue, providing concurrent joint repair. Although we have reported the proteome of neutrophil EVs,^16^ this substantial gene reprogramming prompted us to identify novel EV effectors and, thus, we focused on microRNAs.

### Neutrophil EVs contain RA‐specific microRNAs

Neutrophil EVs from seven healthy donors were tested with a specific microarray chip, containing a total of 2,561 probes against human microRNAs. Single microRNAs were ranked by relative expression levels across donors (Table S6). To control for variability, we plotted the top 100 hits in a heat map (Figure 4A), showing a good degree of consistency for the microRNA populations for the top hits across different EV donors.

Using network visual analytics platform miRNet 2.0 (which collects interactions information from miRTarBase^17^), we constructed a network of EV microRNAs together with their validated microRNA‐target interactions (a cut off was applied to the microRNA subset and only those with expression unit >1 were included in the analysis; Figure 4B). A pathways enrichment analysis highlighted a number of genes common to pathways relevant herein (Figure 4C), including the RA pathway; Wnt signaling, and transforming growth factor beta (TGF‐β) signaling (important for joint and cartilage regeneration). The highlight of specific EV microRNAs that target the 10 hits associated with RA is in Figure 4D. Collectively, these results validate the outcome of the RNA sequencing analysis.

### miR‐455‐3p is one determinant responsible for neutrophil EV chondroprotection

**Figure 5:**
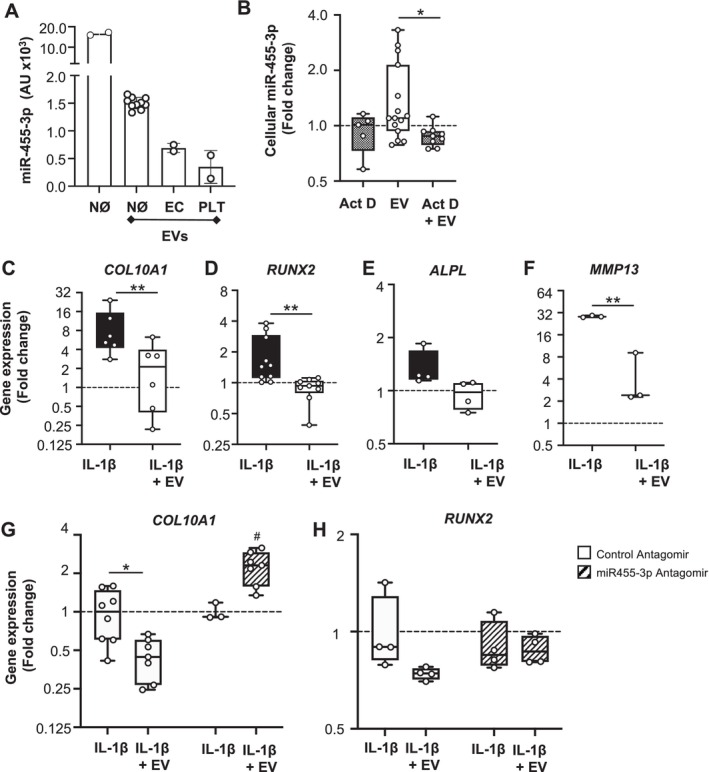
miR‐455‐3p is contained in EVs and exerts anticatabolic properties on chondrocytes. (A) Quantification of miR‐455‐3p in human neutrophils and their EVs by real‐time PCR. EVs (3 × 10^7^) from endothelial cells and platelets were also included for comparison. (B) Human articular chondrocytes were pretreated for 30 minutes with or without actinomycin D, plus or minus neutrophil EVs (3 × 10^7^), and miR‐455‐3p levels were measured after 24 hours. Cells were harvested, and microRNA extracted for reverse transcription and real‐time PCR. Data are reported as fold change from vehicle control (dotted line) *P < 0.05 vs EV Mann‐Whitney test (C–F) Human articular chondrocytes were incubated with IL‐1β (20 ng/mL) with or without healthy donor neutrophil EVs. At 24 hours, real‐time PCR quantification of markers of hypertrophy and/or targets of miR‐455‐3p: *COL10A1* and *RUNX2, ALPL*, and *MMP13* were monitored. Data are reported as fold change from vehicle control (dotted line) **P < 0.01 vs IL‐1β Kruskal‐Wallis test. (G and H) Human articular chondrocytes were treated with combinations of IL‐1β (20 ng/mL) and EVs (3 × 10^7^), with or without presence of a control or specific antagomir. Data are normalized to IL‐1β control. *P < 0.05 vs IL‐1β, #*P* < 0.05 vs IL‐1β+EV following a two‐way ANOVA multiple comparison. Each data point identifies distinct EV donors. ANOVA, analysis of variance; EC, endothelial cell; EV, extracellular vesicle; IL, interleukin; miR, microRNA; NØ, neutrophil; PCR, polymerase chain reaction; PLT, platelet.

A follow up from these in silico data was to demonstrate that it was possible to modulate relevant cell phenotypes using one of the identified microRNAs. We selected miR‐455‐3p, which interacts with genes related to RA (Figure 4C) and is described to regulate chondrocyte hypertrophy.^18^ Comparisons between an equal number of human neutrophils and EVs indicated approximately 10% of miR‐455‐3p content in the vesicles as compared to the parent cell (Figure 5A). EVs isolated from either endothelial cells or platelets contained a one‐third to one‐sixth miR‐455‐3p, when compared to neutrophil EVs (Figure 5A). Addition of neutrophil EVs to human articular chondrocytes elevated cell‐associated miR‐455‐3p levels (Figure 5B). This effect could be blocked when chondrocytes were pretreated with the transcriptional inhibitor actinomycin D (Figure 5B). Functionally, neutrophil EVs inhibited IL‐1β–induced *COL10A1* messenger RNA up‐regulation (Figure 5C), replicating the data of the arthritic joints where delivery of EVs reduced collagen‐X positive cells (Figure S2H). Similar results were obtained for *RUNX2*, a transcriptional driver of chondrocyte hypertrophy, whereby neutrophil EVs reduced *RUNX2* gene up‐regulation induced by IL‐1β (Figure 5D). We also observed counterregulation of another marker of chondrocyte hypertrophy, alkaline phosphatase (*ALPL)* along with down‐regulation of IL‐1β–induced *MMP13* expression by neutrophil EVs, an enzyme involved in cartilage degradation (Figure 5F). An antagomir against miR‐455‐3p reverted the modulatory effect of the EVs on *COL10A1* expression, with a similar trend on *RUNX2* (Figure 5G–H).

### Patient with RA neutrophil‐derived EVs are functionally similar to healthy donor EVs

**Figure 6:**
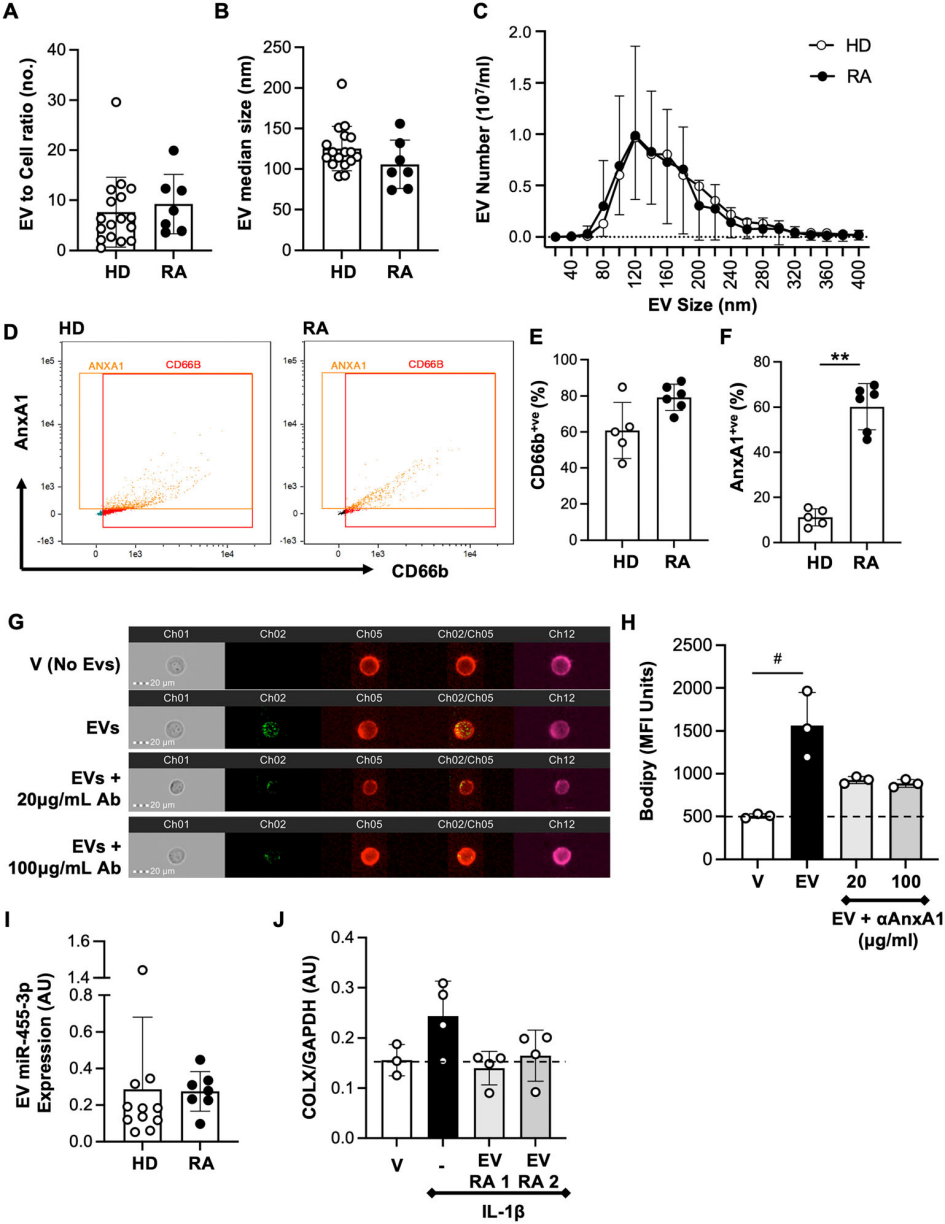
Characterization of RA neutrophil EVs. EVs were obtained from RA and HD neutrophils. (A) Quantification of EV number per cell. (B) Median size distribution of HD and RA neutrophil EVs (C) Average Nanosight profiles for six EV donors per group; mean ± SD (D–F) CD66b and AnxA1 expression on EVs from HD and patients with RA neutrophils. (Left) Representative ImageStream profiles. (Right) Quantitation of positive events (mean ± SD, n = 5–6 preparations per group) *P < 0.05, **P < 0.01 Mann‐Whitney test. (G and H) Uptake of RA neutrophil EVs by human chondrocytes and modulation by anti‐AnxA1 Ab. (Left) Representative ImageStream images showing EV uptake (green) by the whole cells. (Right) Quantitative data (mean ± SD of n = 3 experiments; #*P* < 0.05 Kruskal‐Wallis test with Dunn's multiple comparison). (I) miR‐455‐3p content for 3 × 10^7^ EV per donor, as analyzed by RT‐PCR (n = 7–11). (J) Human articular chondrocytes were stimulated IL‐1β (20 ng/mL) with or without RA EVs (3 × 10^7^) for 24 hours. Collagen type‐X gene expression was quantified by RT‐PCR (mean ± SD of 4 replicates from two donors). Ab, antibody; EV, extracellular vesicle; HD, healthy donor; IL, interleukin; MFI, mean fluorescence intensity; miR, microRNA; RA, rheumatoid arthritis; RT‐PCR, real‐time polymerase chain reaction.

Neutrophil EVs could be utilized as an autologous treatment, in which patient with RA–derived EVs are enriched before direct injection into the joint to aid cartilage repair. Thus, it was important to characterize neutrophil EVs prepared from patients with RA compared with healthy donors (Table S7 and S8). Upon stimulation with TNF‐α, RA neutrophils produced EVs of the same size and number as healthy donor cells (Figure 6A–C). Similarly, RA neutrophils produced vesicles that express CD66b and AnxA1. The proportion of EVs positive for AnxA1 was higher from RA neutrophils (Figure 6D–F; gating outlined in Figure S6). Of interest, RA neutrophil EVs could enter chondrocytes, and their uptake could be partially reversed using an AnxA1 blocking antibody (Figure 6G and H). Finally, miR‐455‐3p levels were comparable in the two populations of EVs (healthy vs RA donor) (Figure 6I) and, similarly to healthy donor EVs, RA EVs could revert the up‐regulation of *COL10A1* gene product in chondrocytes following IL‐1β stimulation (Figure 6J).

## DISCUSSION

We describe that exogenous application of neutrophil EVs results in long‐lasting chondro‐protection and tissue‐protection in the inflamed joint. These effects are associated, at least in part, to the transfer of specific microRNAs from EVs to target cells, which enables transcriptional changes toward remodeling of the cartilage. We propose that neutrophil EVs could be a suitable autologous treatment to regenerate cartilage in inflammatory arthritis and, possibly, other forms of joint damage.

EVs are microstructures packed with several components, able to elicit a variety of biological responses.^10^ Presence of a lipid bilayer protects the EV content from being diluted and preserves physiological concentrations, and expression of specific determinants on the EV surface can facilitate anchoring to target cells, with subsequent transfer of cargo. These physicochemical characteristics explain the longevity of EVs and their activity within the microenvironment. Our own work with neutrophil EVs demonstrated that the EV protein component may vary in relation to the stimulus applied.^16^ Similar changes in EV proteome also occur after stimulation with antineutrophil cytoplasmic antibodies or thrombin,^19^, ^20^ and reflect the neutrophil activation status.

When we mimicked intravascular activation of neutrophils, these cells produced vesicles that activated the endothelium and exerted proinflammatory effects.^21^, ^22^ Contrary, when we mimicked exudate neutrophils, the EVs protected cartilage integrity, an effect in part due to AnxA1.^11^ In addition, these EVs modulated human macrophages toward an anti‐inflammatory phenotype.^23^ Other groups have reported the protective effects of exudate‐like neutrophil EVs^8^ that augment bacterial containment and killing.^24^ The physicochemical characteristics of EVs discussed above justify the interest in these microstructures as potential therapies that target chronic diseases, including arthritis.^25^, ^26^ The possibility to fortify EVs with exogenous products^27^ and incorporate targeting moieties^28^ add to the therapeutic potential of these nanomedicines. We reason that delivery of complex information to target cells and tissues may offer the possibility to effectively modify or revert disease progression. With this in mind, the current study addresses the duration of neutrophil EV's effects and identifies specific molecular determinants responsible for this pharmacology.

In the STIA model, the combined outcomes of experiments with neutrophil EVs (prophylactic and therapeutic protocols) reveal potent and long‐lasting protection on cartilage. Specifically, local injection of EVs protects structural integrity of the cartilage, a macroscopic outcome consequent to prevention of collagen type‐II loss together with decreased proportion of collagen type‐X positive chondrocytes. The latter effect indicates an inhibition of chondrocyte hypertrophy, which drives cartilage breakdown in OA, in which loss of structural integrity within the joint is a primary endpoint.^29^, ^30^, ^31^ Such an effect on chondrocyte phenotype prompts us to propose that neutrophil EVs do not simply inhibit inflammation but can actually play active regenerative and anticatabolic roles through a direct effect on the chondrocyte. This conclusion is reinforced by the efficacy of delivery of neutrophil EVs during on‐going aggressive joint arthritis. Analysis of osteophyte formation by histology revealed that EV delivery did not affect bone remodeling within the joint. EV‐mediated regenerative effect seems to be articular cartilage specific because it does not drive detrimental terminal differentiation and calcification of the tissue, which is, in fact, inhibited. It is plausible that therapeutic application of neutrophil EVs may extend to other diseases, such as OA, in which the damage is mechanically driven. Data obtained with the humanized CD1^nu/nu^ in vivo model corroborate this suggestion, with a direct effect of neutrophil EVs on transplanted human chondrocytes in settings in which inflammation is minimal. Future studies in experimental OA will challenge this proposition though this potential utilization has already been proposed.^32^, ^33^ We have confidence that the pharmacological effects of neutrophil EVs is sex independent. Although the STIA experiments performed herein were in male mice, we have previously reported that neutrophil EVs are effective in female mice during antigen‐induced arthritis,^28^ along with our new data demonstrating their efficacy in the female cartilage explant model.

In our in vivo experiments, EVs produced a modest anti‐inflammatory action without antinociception. The latter notion was corroborated by lack of modulation of calcitonin‐gene–related peptide expression in the dorsal root ganglia, a major site for chronic pain signaling.^34^, ^35^ However, these experimental conditions are not ideal to study anti‐inflammatory and antinociceptive effects, in view of the intra‐articular route of administration in a polyarthritis model; therefore specific experimental protocols need to be applied to specifically address this.

To acquire novel information on how neutrophil EVs impact on joint disease progression, we ran RNA sequencing analysis in naive versus diseased joint, comparing also diseased joint with or without EV treatment. When focusing on disease versus naive analysis, the gene with the greatest fold increase in disease was *Kcne4*, encoding for potassium voltage‐gated channel subfamily E member 4, which has been associated with chondrocyte subtypes defective to form a cartilage matrix.^36^ In the second comparison, differentially expressed genes that were unique to EV treatment (or counterregulated by EVs against disease alone) clustered with signal transduction, indicating a role for EV in mediating cellular processes rather than driving extracellular matrix maintenance per se. This observation is aligned with the multiple signals that EVs can convey. In this analysis, *Heyl*, encoding a basic helix‐loop‐helix–type transcription factor, showed the greatest fold increase; this gene product is described to mediate the effects of notch signaling and regulate cartilage homeostasis.^37^ In addition, membrane transporters such as *Kcnk1*, a two‐pore domain K^+^ channel important for regulation of chondrocyte membrane potential,^38^ was up‐regulated. The most strongly down‐regulated gene was *Slc7a10*, which encodes for the Asc‐type amino acid transporter 1, again already associated with OA.^39^ Although these exemplar genes may not represent the classic panel targeted when developing therapeutic approaches to tackle cartilage breakdown, they could be used as markers to guide the development of novel chondroprotective therapies. Together with the pathways and cellular processes associated with treatment with EVs, such as the WNT pathway, our RNA sequencing data suggest a change in cell phenotype indicating regeneration and remodeling of the tissue, rather than cartilage matrix maintenance. Further investigations are required to elucidate the specific downstream mechanism of the EV effect in the cartilage tissue, the timing of these events, and also to understand the contribution of acute inflammation to these cartilage remodeling processes.

A major obstacle to the development of effective treatments of the diseased cartilage is represented by its anatomy. Chondrocytes are distributed within their lacunae, scattered across a dense and negatively charged extracellular matrix, without vasculature; as such these cells are difficult to reach: antibodies and antibody fragments may partly penetrate the cartilage matrix.^40^, ^41^ Using organ culture approaches and damaging cartilage with proinflammatory cytokines, we demonstrated that neutrophil EVs reach chondrocytes and deliver their content into the lacunae.^11^ Such a property appears specific for neutrophil EVs because it is not replicated by monocytes EVs, and it could be secondary to specific chemotactic cues released by the chondrocytes. Exosomes with a protective payload can also reach cartilage in vivo.^42^

Next, we focus on the determinants within neutrophil EVs responsible for these biological effects. In line with previous work,^11^ AnxA1 exposure on membrane‐borne EVs facilitates docking and uptake by chondrocytes, a mechanism also reported for T cells^43^ and macrophages.^23^ Endothelial cells take up EVs through an AnxA1‐mediated interaction with phosphatidylserine, ultimately preventing apoptosis.^44^ Notwithstanding the role of EV AnxA1, we reasoned that other factors ought to explain the long‐lasting effects of neutrophil EVs presented herein. The data on modulation of gene expression in the STIA joint and the duration of pharmacological efficacy, prompted us to run an array of microRNAs on neutrophil EV preparations.

It is accepted that EVs contain microRNAs that mediate a variety of their biological effects (reviewed in Oggero et al^45^). Our analysis with the publicly available dataset miRTarBase (which reports validated microRNA‐target interactions) identified major interactions between the top expressed microRNAs present in neutrophil EVs, and specific pathways relevant to our study. In particular, pathways associated with joint development and cartilage remodeling like Wnt, TGF‐β signaling (corroborating our RNA sequencing data), and RA. As a proof of concept, we focus on miR‐455‐3p because it was highlighted in our analysis and described to regulate chondrocyte hypertrophy,^46^ but also appreciating this is not the only active microRNA found within the neutrophil EVs.

We detect high levels of miR‐455‐3p in neutrophils and in their vesicles. Moreover, neutrophil EVs containing miR‐455‐3p produce a positive feedback on endogenous chondrocyte miR455‐3p expression in chondrocyte cultures, with an anticatabolic outcome. In a number of different in vitro settings, miR‐455‐3p expression increases as cells undergo chondrogenic differentiation,^47^, ^48^, ^49^ in particular in the early and middle phases of chondrogenesis, but then drops off as cells become hypertrophic. In keeping with this, miR‐455‐3p may play a direct role in inhibiting late‐stage differentiation and hypertrophy through inhibition of *Runx2*, a major transcription factor driving this function.^46^ Of note, miR‐455‐3p is also expressed during OA^50^, ^51^; hence it is associated with disease pathogenesis. This evidence and its regulation of TGF‐β signaling^51^ highlights an important role in joint remodeling and homeostasis. Our results add to this body of evidence, confirming the important role that microRNA play in tissue regeneration within the joint and supporting their role as important contributors for mediating EV function.

Finally, to propose potential application of an autologous EV‐based therapy, it was important to test EVs from patients suffering from RA. Production of EVs from neutrophils of RA blood yielded vesicles that are physically undistinguishable from those generated from age‐matched healthy control volunteers. RA neutrophil EVs contain miR‐455‐3p to a similar extent to healthy control EVs, and application to chondrocytes in vitro elicited similar anticatabolic responses.

In conclusion, we provide evidence for long‐lasting reparative effects of neutrophil EVs on cartilage in settings of experimental arthritis. Coupled to the observation that large amounts of neutrophil EVs are present in RA synovial fluids^11^ and that circulating neutrophils from patients with RA can generate EVs with appropriate cargo (eg, AnxA1; miR‐455‐3p), we propose that innovative therapeutic approaches for joint disease could rely on natural or semisynthetic^52^ vesicles as nanomedicines in the near future.

## AUTHOR CONTRIBUTIONS

All authors contributed to at least one of the following manuscript preparation roles: conceptualization AND/OR methodology, software, investigation, formal analysis, data curation, visualization, and validation AND drafting or reviewing/editing the final draft. As corresponding author, Dr Norling confirms that all authors have provided the final approval of the version to be published, and takes responsibility for the affirmations regarding article submission (eg, not under consideration by another journal), the integrity of the data presented, and the statements regarding compliance with institutional review board/Helsinki Declaration requirements.

## Supporting information


Disclosure form

**Appendix S1:** Supplementary methods

**Appendix S2:** Supplementary Information

**Table S1:** Supplementary Tables
