# Supplementary material for: Molecular Determinants of Neutrophil Extracellular Vesicles That Drive Cartilage Regeneration in Inflammatory Arthritis
Source: Arthritis Rheumatol. 2024 Aug 16;76(12):1705–18. doi: 10.1002/art.42958 (PMC11605269; doi:10.1002/art.42958)
Supplement: Supplementary file 2 — Appendix S1: Supplementary methods [file ART-76-1705-s002.docx]

Supplementary methods:

ImageStream™ analysis for EV surface markers

EV acquisition on the IS_X_ MKII imaging cytometer was slow flow rate, 60x magnification and any excitation lasers and the 785nm (scatter) laser turned on at full power. The remove beads function was unticked and flow speed stabilised prior to EV acquisition. EVs were analysed and counted using fluorescence triggering as described (1). EVs were labelled with 50 μM BODIPY maleimide fluorescein or BODIPY Texas-Red (Life Technologies, USA; Cat#A-5770, RRID:AB2536193), or labelled with either anti-CD66b-PE (2 μg/ml; 6/40c, Biolegend) or anti-AnxA1-PB (clone 1B; 1 μg/ml; generated in-house) (2). Data were analysed using FlowJo^TM^ Software with ~20,000 events acquired per sample. Gating for AnxA1 and CD66b positive EVs was established using fluorescence minus one (FMO) controls (Figure S6).

ImageStream™ analysis for EV uptake

Neutrophil EVs (1x10^7^) were stained with BODIPY FITC for 20min and incubated with human articular chondrocytes in 6-well plates for 6h, 5% CO_2_ at 37ºC. In some experiments EVs were pre-treated for 30min with anti-AnxA1 antibody (100ng/ml; clone 1B) and washed prior to incubation with cells. Cells were washed twice with PBS, detached with Accutase, re-suspended in 50µl, stained with CD40-Pe/Cy7 (Clone HB14; Biolegend). IS_X_ MKII imaging cytometer was used to detect presence of EVs inside chondrocytes with slow acquisition rate.

Western blotting

Neutrophil-EV samples were lysed in RIPA buffer (ThermoFisher; cat. no. #89901) with 1:100 protease and phosphatase inhibitors (ThermoFisher; cat. no. #1861281). EV solutions were vortexed and incubated at 4°C for 20 min. EV concentration was determined by micro-BCA (Boster Bio; cat. no. #AR1110) with standards prepared in 10% v/v RIPA lysis buffer with 0.22-μm filtered deionised H_2_O. Neutrophil-EV (4 μg) solutions were mixed with 25% v/v Laemmeli buffer (Thermo Fisher; cat. no. #NP0007) with 1% v/v Dithiolthereitol (DTT). Sample volume was made up to 15 μl with 0.22 μm filtered deionised H_2_O and denatured at 95°C for 10 min. Samples were run on a 10% w/v poly-acrylamide gel at 120V for 90 min and transferred to Immobilon membrane (Sigma; cat. no. #IPVH00010) using a semi-dry transfer at 200 milliAmps for 35 min. Membranes were blocked in 5% w/v milk-TBS-T for 1 hour at room termperature and primary antibodies (in 5% w/v milk-TBS-T) were incubated overnight at 4°C. Then, membranes were washed and incubated with the appropriate secondary horseradish peroxidase (HRP)-conjugated antibody for 1 hour at room termperature and detected using Immobilon Forte Western Blotting HRP substrate. To re-probe for additional markers, membranes were stripped (pH 2.2 stripping buffer) for 20 min at room termperature. Membranes were then washed and protocol repeated. Primary and secondary antibody used in the study are included below.

| **Protein of interest** | **MW (kDa)** | **Dilution** | **Host** | **Company** | **Cat no.** |
| --- | --- | --- | --- | --- | --- |
| **Annexin A1** | 38 | 1:2000 | Rabbit | Abcam | ab214486 |
| **Flotillin-1** | 49 | 1:1000 | Rabbit | Cell Signalling Technology | 18634 |
| **CD63** | 25 | 1:10000 | Mouse | Invitrogen | 10628D |
| **CD9** | 24 | 1:5000 | Rabbit | Invitrogen | MA5-31980 |
| **Calnexin** | 90 | 1:2000 | Rabbit | CST | 2679S |
| **HRP-conjugated anti-rabbit Ig** | - | 1:2000 | Goat | Dako | P044801 |
| **HRP-conjugated anti-mouse Ig** | - | 1:2000 | Goat | Dako | P044701 |

Immunogold labelling and Transmission Electron Microscopy (TEM) of EVs. Briefly, TEM copper grids (400 mesh, Agar Scientific, Essex, UK) were pre-coated with 1% formvar (Agar Scientific) solution prepared in chloroform as detailed (3). EV suspension (1x10^6^/10µl) was applied directly onto TEM grids prior to washing and fixation in 4% paraformaldehyde, 4°C for 20min. After quenching with 20mM glycine, grids were incubated with anti-AnxA1 mAb (5ng/ml; clone 1B produced in house), 1 hour at room temperature. For primary antibodies raised in mice, a rabbit-anti-mouse antibody was applied (Dako, Glostrup, DK) for 45 min. All grids were incubated with Protein Gold A (PGA; 10-nm sized gold, UMC Utrecht, NL), before washing and fixing in 1% glutaraldehyde. Grids were finally stained in 2% uranyl acetate for 2 min. A JEOL 1400+ TEM (Tokyo, Japan) equipped with an AMT XR16 CCD camera (AMT, Massachusetts, USA) to acquire images with magnification between x8,000 and x20,000.

Histological analysis of tissue

Following dissection, joints were fixed for 24h in 4% paraformaldehyde, then decalcified (10% EDTA buffer for 2 weeks). Joints were processed and paraffin-embedded for sectioning at 5-μm. Cartilage nodules from the ectopic cartilage formation assay were embedded in OCT for cryo-sectioning.

For proteoglycan analysis, sections were stained with either 0.2% Safranin O or 0.1% Toluidine blue, both in acetate buffer at pH 4 and 5, respectively. All sections within a single analysis were stained and imaged together. Intensity of staining was measured by densitometry; the cartilage surfaces were isolated and the extracted value normalised for the subchondral bone within each section, to control for any intra-section variability using ImageJ software.

To measure structural breakdown within the cartilage the Osteoarthritis Research Society International (OARSI) scoring system was used. A score of 0-6 was applied per compartment (medial and lateral of the tibial and femoral surfaces; and summed altogether), with 6 representing most severe (4). Inflammation and bone resorption scores are outlined in Supplementary Figure S3 (adapted from (5, 6)). Osteophyte formation was scored on absence or presence of ≥1 osteophyte/joint.

For immuno-histochemical analysis, antigen unmasking was performed with pepsin digestion (for paraffin-embedded samples) and sections stained for collagen type-I (EMD Millipore MAB8887) then collagen type-X (eBioscience; Ref 41.9771-82). Image analysis was performed with ImageJ software.

RNA extraction and analysis by quantitative real-time PCR

Total RNA was extracted from cells using TrizolTM reagent (Thermofisher). Reverse transcription was conducted with SuperScript™III Reverse Transcriptase (Thermofisher) and qPCR analysis run with HotStarTaq DNA Polymerase and supplied buffer (Qiagen). The total PCR run mix was made up with the following additional components: template cDNA, Quantitect primers (Qiagen; *GAPDH*: QT01192646; *COL10A1*: QT00096348; *RUNX2*: QT00020517; *MMP13: QT00001764*) dNTPs (Promega), SYBR green (Invitrogen) and Rox reference dye (Invitrogen). For miRNA analysis miScript II RT kit (Qiagen) was used to obtain cDNA, followed by miScript SYBR Green PCR kit (Qiagen) using primer set for mir-455-3p (MS00009744) and reactions run per manufacturer’s instructions. All qPCRs were run on ABI QuantStudio™ 7 Flex Real-Time PCR System. Analysis was performed using the relative quantification method and reported as relative quantity of target gene normalised to housekeeping (*GAPDH*).

For RNA sequencing, mouse joints were dissected and cartilage isolated along with the underlying subchondral bone. Following RNA extraction using Trizol^TM^, samples were processed and analysed by GenXPro GmbH (Goethe University, Germany). MACE-Seq libraries from the extracted RNA were prepared using the MACE-Seq Kit (GenXPro) according to the manufacturer’s instructions. The resultant samples were then sequenced on an Illumina NextSeq500 machine with at least 5M reads per sample. A total of 30,882 features were detected across all samples. Data were normalised using the transcripts per kilobase million (TMP) method. P-value and FDR were calculated using two-tail paired t-test and Benjamini & Hochberg method respectively. To remove background noise, features with an average <10 normalised counts in at least one group were removed. Genes with p<0.05 in at least one of the comparisons (Disease *vs.* Naive or EV-treated *vs.* Contralateral vehicle-treated) were selected, resulting in a final working list of 5,231 genes. Functional analysis was conducted using Panther Classification System v12.0. Raw data was deposited in the public repository Gene Expression Omnibus (GEO Accession Number: GSE194152).

**References:**

1. Headland SE, Jones HR, D'Sa AS, Perretti M, and Norling LV. Cutting-edge analysis of extracellular microparticles using ImageStream(X) imaging flow cytometry. *Sci Rep.* 2014;4:5237.

2. Pepinsky RB, Sinclair LK, Dougas I, Liang CM, Lawton P, and Browning JL. Monoclonal antibodies to lipocortin-1 as probes for biological function. *FEBS Lett.* 1990;261(2):247-52.

3. Oggero S, de Gaetano M, Marcone S, Fitzsimons S, Pinto AL, Ikramova D, et al. Extracellular vesicles from monocyte/platelet aggregates modulate human atherosclerotic plaque reactivity. *J Extracell Vesicles.* 2021;10(6):12084.

4. Glasson SS, Chambers MG, Van Den Berg WB, and Little CB. The OARSI histopathology initiative - recommendations for histological assessments of osteoarthritis in the mouse. *Osteoarthritis Cartilage.* 2010;18 Suppl 3:S17-23.

5. Bendele A, McAbee T, Sennello G, Frazier J, Chlipala E, and McCabe D. Efficacy of sustained blood levels of interleukin-1 receptor antagonist in animal models of arthritis: comparison of efficacy in animal models with human clinical data. *Arthritis Rheum.* 1999;42(3):498-506.

6. Pettit AR, Ji H, von Stechow D, Muller R, Goldring SR, Choi Y, et al. TRANCE/RANKL knockout mice are protected from bone erosion in a serum transfer model of arthritis. *Am J Pathol.* 2001;159(5):1689-99.
