# Supplementary material for: Molecular Determinants of Neutrophil Extracellular Vesicles That Drive Cartilage Regeneration in Inflammatory Arthritis
Source: Arthritis Rheumatol. 2024 Aug 16;76(12):1705–18. doi: 10.1002/art.42958 (PMC11605269; doi:10.1002/art.42958)
Supplement: Supplementary file 3 — Appendix S2: Supplementary Information [file ART-76-1705-s003.pdf]

Supplementary Information

Molecular determinants of neutrophil extracellular vesicles that drive cartilage regeneration in inflammatory arthritis

Bethan L Thomas[1], Trinidad Montero-Melendez[1,2], Silvia Oggero[1,3], Magdalena K Kaneva[1,2] David Chambers[3], Andreia L Pinto[4], Alessandra Nerviani[1], Davide Lucchesi[1], Shani Austin-Williams[1], Mohammed Hussain[1], Costantino Pitzalis[1,2], Benjamin Allen[3], Marzia Malcangio[3], Francesco Dell'Accio[1,2], Lucy V Norling[1,2], Mauro Perretti[1,2].

Figure S1. Neutrophil extracellular vesicle characterization.
Figure S2. Dose optimization for neutrophil EVs in inflammatory arthritis.
Figure S3. Scoring guidelines for inflammatory and bone erosion scores.
Figure S4. Analysis of nociception in arthritic mice.
Figure S5. Neutrophil EV uptake by human chondrocytes.
Figure S6. Neutrophil EV gating strategy by ImageStream™

Table S1-S6. Separate Excel files

Table S1. Gene differentially expressed between arthritic and naïve joints (RNA seq).
Table S2. Gene differentially expressed between EV-treated and vehicle-treated joints of arthritic mice (RNAseq).
Table S3. Genes significantly regulated and unique to arthritic and naïve joints comparison (not regulated in EV-treated vs vehicle-treated joints of arthritic mice).
Table S4. Genes significantly regulated and unique to EV-treated vs vehicle-treated joints of arthritic mice (not regulated in arthritic vs naïve joints comparison).
Table S5. Comparison of opposite gene modulation between arthritis and arthritis plus EVs.
Table S6. miRNA analyses in neutrophil EVs: ranked list.
Table S7. Demographic information of healthy donors.
Table S8. Demographic information of rheumatoid arthritis patients.

Supplementary Methods

Figure S1

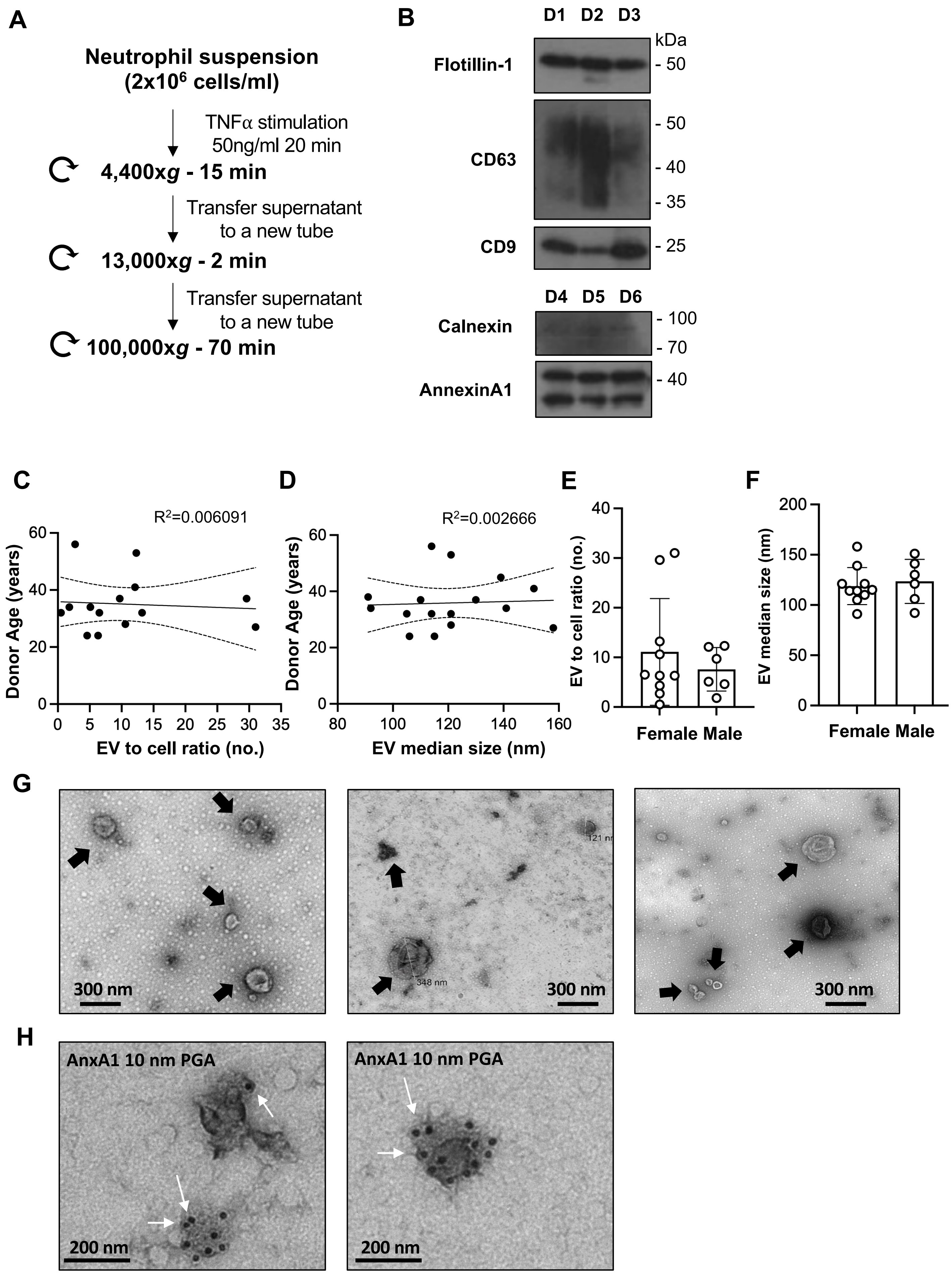
A
Neutrophil suspension
(2x10^6 cells/ml)
TNFα stimulation
50ng/ml 20 min
4,400xg - 15 min
Transfer supernatant
to a new tube
13,000xg - 2 min
Transfer supernatant
to a new tube
100,000xg - 70 min
B
D1 D2 D3
kDa
Flotillin-1
- 50
CD63
- 50
- 40
- 35
CD9
- 25
D4 D5 D6
Calnexin
- 100
- 70
AnnexinA1
- 40
C
R2=0.006091
Donor Age (years)
EV to cell ratio (no.)
D
R2=0.002666
Donor Age (years)
EV median size (nm)
E
EV to cell ratio (no.)
Female Male
F
EV median size (nm)
Female Male
G
300 nm
121 nm
348 nm
300 nm
300 nm
H
AnxA1 10 nm PGA
200 nm
AnxA1 10 nm PGA
200 nm

Figure S1. Neutrophil extracellular vesicle characterization. Extracellular vesicles (EVs) were produced from blood-borne isolated neutrophils following 20 min stimulation with 50ng/ml TNF-a. **A) I**solation protocol for neutrophil EVs. **B)** Western blots of human neutrophil-EVs from 6 individual donors, isolated by differential centrifugation. Antibodies were used to detect flotillin-1 (49kDa), CD63 (~30-60kDa), CD9 (~29kDa), annexinA1 (~35 kDa) and calnexin (~67–90kDa) CD81 was not detected on these EV (data not shown). **C,D)** Correlation comparing healthy donor age against number of EVs produced per neutrophil or against EV size (nm). **E, F)** Comparison of female *vs.* male healthy donor EVs with respect to number produced per neutrophil as well as median size in nm. Mean ± SD. **G)** Representative transmission electron microscopy images of neutrophil EVs analysed by electron microscopy (arrows, EVs of different morphology). **H)** Annexin A1 (AnxA1)-gold labelled EVs with 10 nm Protein Gold A (PGA; white arrow). Representative from three distinct preparations.

Figure S2

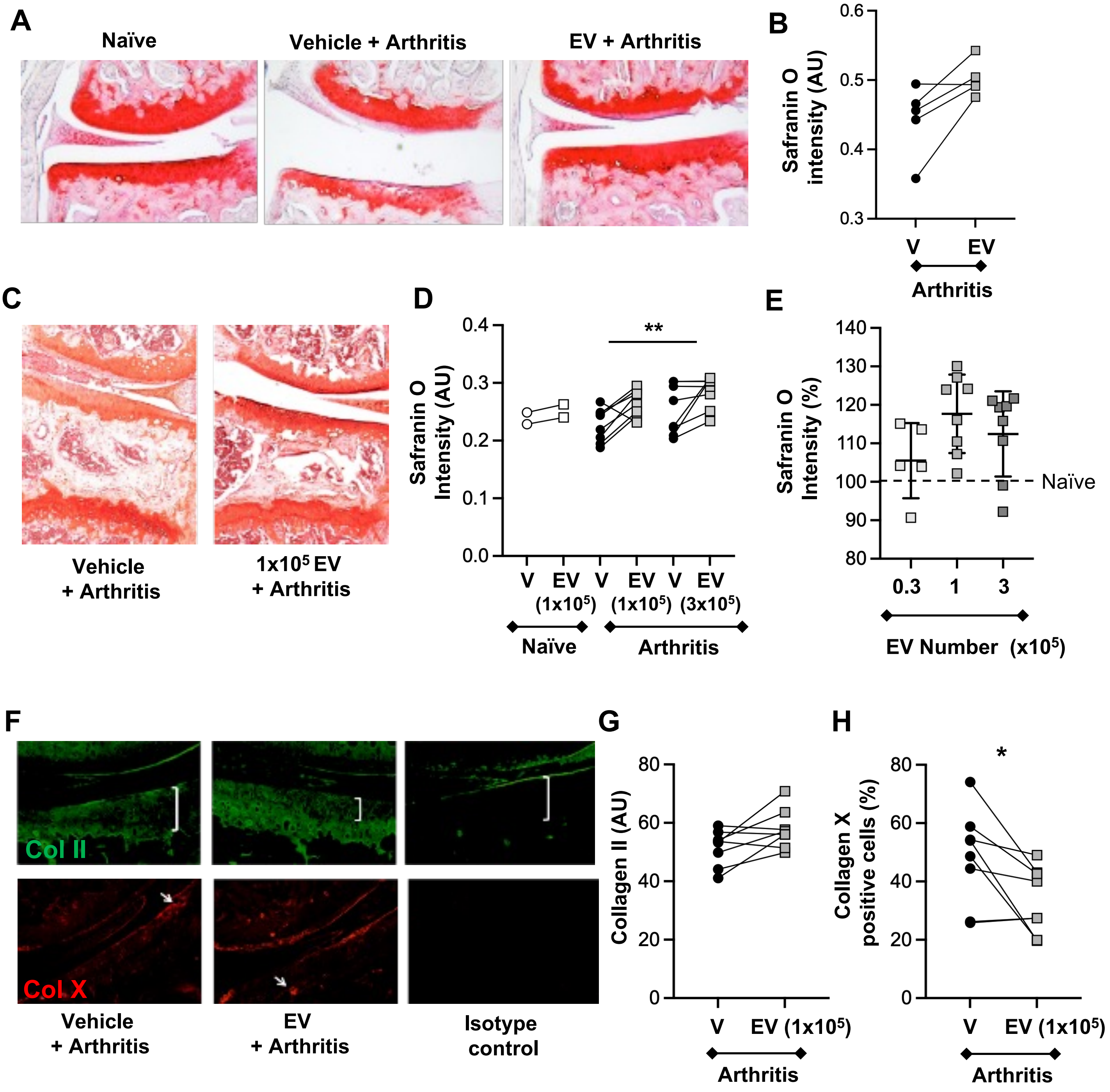

Figure S2. Dose optimization for neutrophil EVs in inflammatory arthritis. K/BxN arthritis was induced in mice with 100 µl serum injections on day 0 and day 2. Neutrophil EVs were injected into the knee articular space on day 3 and cartilage staining quantified 48 hours later (Day 5). **A)** Representative images of safranin O staining used to measure proteoglycan content for naïve, arthritic and EV (0.3x10^5)-treated arthritic mice. **B)** Paired analysis of Safranin O staining. Data are mean from 5 mice per group. **C-H)** Arthritis conducted as above but terminated on Day 10. C) Representative Safranin O staining images of arthritic mouse knees after treatment with vehicle or 1.0x10^5 EVs. **D)** Paired quantification of Safranin O staining across two doses of EV, as compared to naïve mouse joints (no arthritis). Each point represents a different knee pairing. Data were modelled using a two-way ANOVA where treatment and dose were entered as predictors, with a random intercept per mouse to account for matching between knees **P<0.01 two-way ANOVA for treatment. A QQ plot was assessed to ensure that residuals approximated towards a normal distribution **E)** Data as in D but expressed as percentage of respective contra-lateral vehicle-treated knees (control knee). **F)** Staining for collagen type II and collagen type X on day 10 with joint treated with 1.0x10^5 EV on day 3. **G,H)** Quantification of the intensity of collagen II staining within the extracellular matrix and number of collagen type X positive cells, expressed as a percentage of total number of cells (DAPI+). *P<0.05, Wilcoxon matched-pairs signed rank test.

Figure S3

	Inflammatory score
0	Normal joint with no inflammation
1	Mild synovitis with no effect to cartilage
2	Clear hyperplasia with synovial infilltrate and cartilage errosions
3	Strong cellular infilltration and clear errosions to bone and cartilage leading to joint disruption

	Bone resorption score
0	Normal joint with no errosions
1	Minimal - few areas of resorption, not visible on low magnification
2	Mild - more numerous areas of resorption, not visible on low magnification
3	Moderate - obvious resorption in trabecular and cortical bone but without full thickness defects
4	Marked - few full thickness defects in the cortical bone often with distortion of the surface profile
5	Severe - numerous full thickness defects in the cortical bone with severe distortion of the surface profile

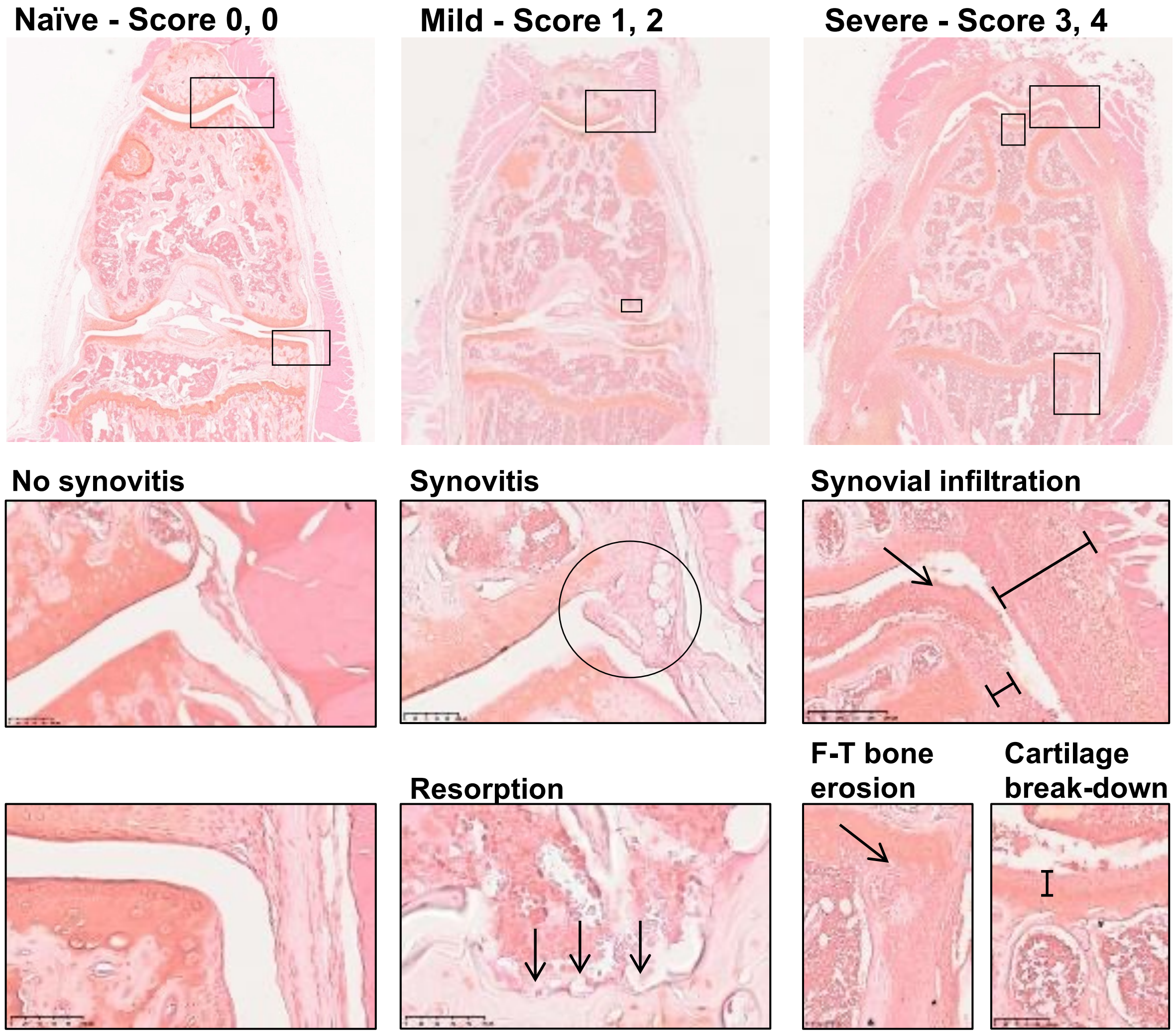

Figure S3. Scoring guidelines for inflammatory and bone erosion scores (adapted from (57, 58)). Representative images of naïve, mild and severe scores in the knees of KBxN arthritic mice at day-30 post-disease (100 µl serum injections intraperitoneally, on day 0, 2, 13 and 24). Black boxes in overview images, denote areas shown below to magnify disease phenotypes. Circle, focus on synovitis. Markers and arrows indicate thickening of the synovial lining and pannus formation within synovial thickening inset box, site of erosion and cartilage surface, in bottom right insets**.**

Figure S4

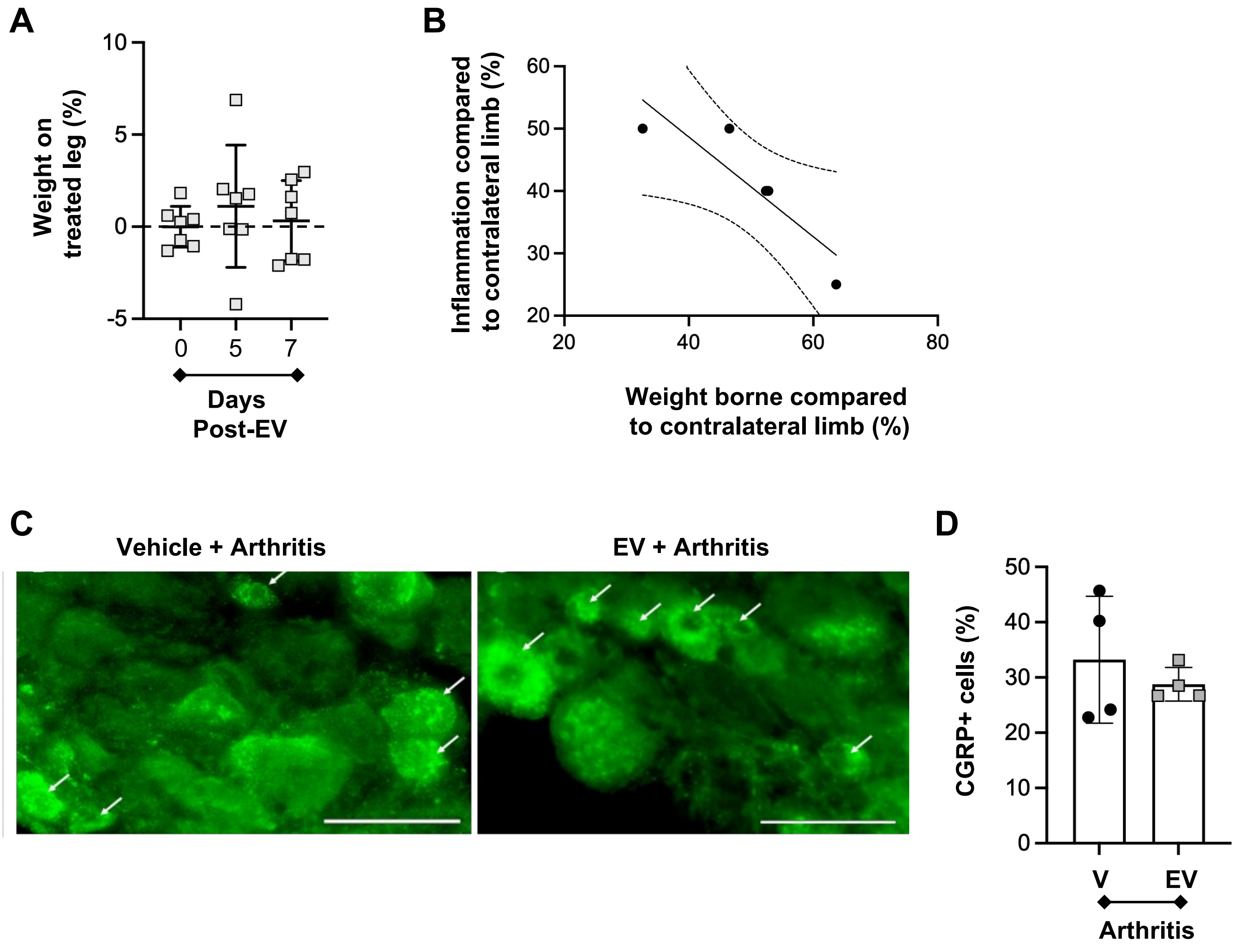

Figure S4. Analysis of nociception in arthritic mice. K/BxN arthritis was induced in mice with 100 µl serum injections on day 0 and day 2. Neutrophil EVs ($0.3x10^5$) were injected into the knee articular space on Day 3. A) Weight bearing between hind limbs measured at day 5 and day 7; values expressed as a percentage of contra-lateral control. B) Correlation of incapacitance data in panel A and relative arthritis disease score for each mouse (taking 100% the peak value). C) Representative images for calcitonin-gene related peptide (CGRP) immunoreactivity in lumbar dorsal root ganglion neurons as measured on Day 10 of arthritis (7 days after $1.0x10^5$ EV treatment). Arrowheads denote dorsal root ganglia cell bodies that express high CGRP-immunoreactivity. Scale bars, 50-µm. D) Quantitative data for percentages of DRG cell bodies that express high CGRP immunoreactivity. Data are mean ± SD, n=4.

Figure S5

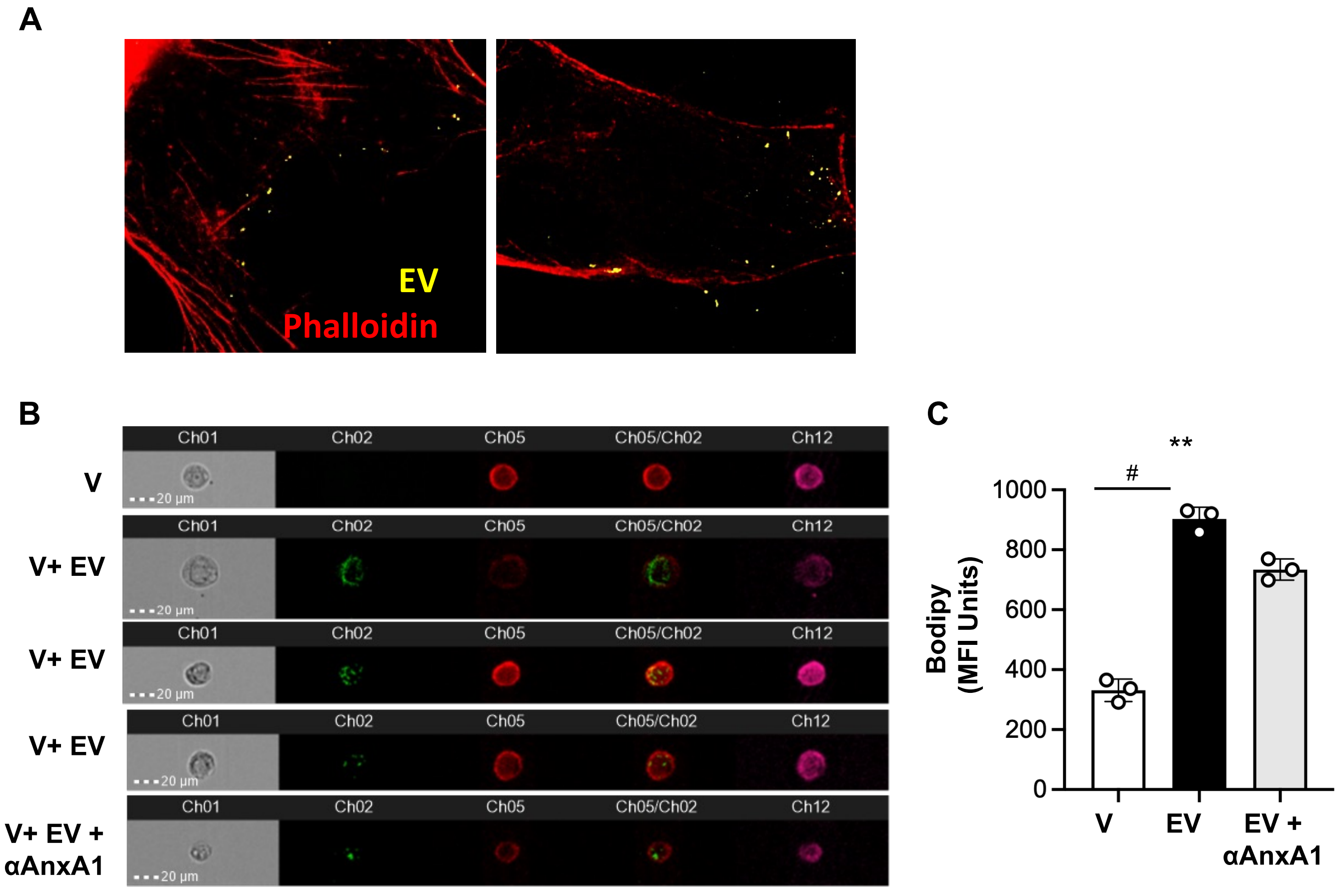

Figure S5. Neutrophil EV uptake by human chondrocytes. Neutrophil EVs were added to C28/I2 chondrocyte cell for 6 hours. A) Immunostaining images of C28/I2 cells (phalloidin; red) and EVs (Bodipy; yellow). B) ImageStream images of C28/I2 cells (phalloidin; red) and neutrophil EVs (Bodipy; green) from a separate experiment. In some cases, an anti-AnxA1 blocking antibody was added. C28/I2 cells harvested and stained for CD44 prior to ImageStream™ analysis. Bar graphs present quantitative data for Bodipy intensity staining within the cells. Data are mean ± SD of three distinct preparations and incubations (Kruskal Wallis test ** $p<0.01$ with Dunns multiple comparison # $p<0.05$).

Figure S6

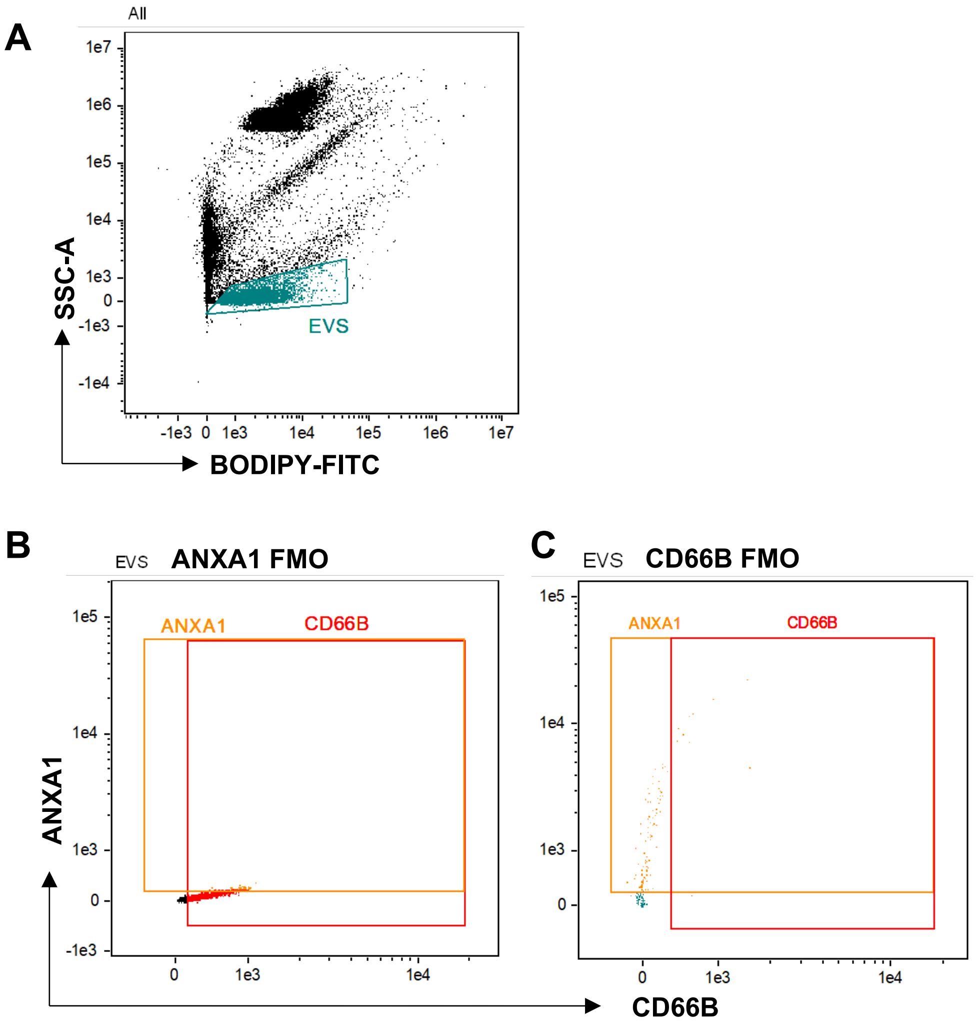

Figure S6. Neutrophil EV gating strategy by ImageStream^{TM}.
Neutrophil EVs were prepared from healthy donors and analysed using ImageStream as detailed in the methods. EVs were gated according to BODIPY-FITC fluorescence and low side scatter (A). Gating for ANXA1 (B) and CD66B (C) positive EVs was established using fluorescence minus one (FMO) controls. Fully stained samples are shown in Figure 6.

Table S7. Demographic information of healthy donors.

EV ID	Age	Gender	Disease status
**HD1**	38	F	Healthy
**HD2**	34	M	Healthy
**HD3**	32	F	Healthy
**HD4**	32	F	Healthy
**HD5**	45	F	Healthy
**HD6**	34	M	Healthy
**HD7**	24	M	Healthy
**HD8**	28	F	Healthy
**HD9**	24	F	Healthy
**HD10**	41	M	Healthy
**HD11**	32	F	Healthy
**HD12**	56	F	Healthy
**HD13**	37	F	Healthy
**HD14**	53	M	Healthy
**HD15**	37	M	Healthy
**HD16**	24	F	Healthy
**HD17**	34	F	Healthy

Table S8. Demographic information of rheumatoid arthritis patients.

Patient ID	Age	Gender	RF status	Anti-CCP status
**RA4**	32	F	Positive	Positive
**RA6**	73	F	Positive	Positive
**RA7**	41	M	Positive	Positive
**RA8**	45	F	Negative	Positive
**RA9**	44	F	Positive	Positive
**RA10**	51	F	Negative	Positive
**RA11**	68	F	Negative	Negative
**RA12**	39	F	Positive	Positive
**RA13**	35	M	Negative	Positive
**RA14**	52	F	Positive	Positive
